# Different Levels of Therapeutic Strategies to Recover the Microbiome to Prevent/Delay Acute Lymphoblastic Leukemia (ALL) or Arrest Its Progression in Children

**DOI:** 10.3390/ijms25073928

**Published:** 2024-03-31

**Authors:** Tommaso Silvano Aronica, Miriam Carella, Carmela Rita Balistreri

**Affiliations:** 1 Complex Operative Unit of Clinical Pathology, ARNAS Civico Di Cristina e Benfratelli Hospitals, 90127 Palermo, Italy; tommasosilvano.aronica@arnascivico.it (T.S.A.); miriam.carella@edu.unito.it (M.C.); 2 Cellular, Molecular and Clinical Pathological Laboratory, Department of Biomedicine, Neuroscience and Advanced Diagnostics (Bi.N.D.), University of Palermo, 90134 Palermo, Italy

**Keywords:** acute lymphoblastic leukemia (ALL), microbiome, GM, therapeutic strategies

## Abstract

Changes in the components, variety, metabolism, and products of microbiomes, particularly of the gut microbiome (GM), have been revealed to be closely associated with the onset and progression of numerous human illnesses, including hematological neoplasms. Among the latter pathologies, there is acute lymphoblastic leukemia (ALL), the most widespread malignant neoplasm in pediatric subjects. Accordingly, ALL cases present a typical dysfunctional GM during all its clinical stages and resulting inflammation, which contributes to its progression, altered response to therapy, and possible relapses. Children with ALL have GM with characteristic variations in composition, variety, and functions, and such alterations may influence and predict the complications and prognosis of ALL after chemotherapy treatment or stem cell hematopoietic transplants. In addition, growing evidence also reports the ability of GM to influence the formation, growth, and roles of the newborn’s hematopoietic system through the process of developmental programming during fetal life as well as its susceptibility to the onset of onco-hematological pathologies, namely ALL. Here, we suggest some therapeutic strategies that can be applied at two levels of intervention to recover the microbiome and consequently prevent/delay ALL or arrest its progression.

## 1. Introduction

Leukemia represents a typical and heterogeneous group of hematological neoplasms that have the characteristics of clonal dominance of hematopoietic stem cells arrested in the development and maturation of multipotent progenitors [1]. This pathological condition is highly prevalent in children under 15 years of age (accounting for 30% of all cancers), where it represents the most common form of childhood malignant neoplasm. Precisely, forms of leukemia in children mainly consist of acute lymphoblastic leukemia (ALL) and acute myeloid leukemia (AML). Children within the age range of 2–4 years are the most common ALL cases and show an overall survival (OS) of above 90% [2]. ALL is characterized by a substantial proliferation of lymphoblasts and declined levels of circulating mature cells, which are responsible for the development of unfunctional bone marrow and consequent failure syndrome [3,4,5]. ALL may be of two forms: the first, with a prevalence of 80% in children, causing alterations in the B cell lineage, and the second, which is uncommon and is characterized by affecting T cells. The major ALL complication, which can lead to mortality, is the involvement of the central nervous system (CNS), with the development of typical symptoms, including the loss of balance, headache, fainting, nausea, or, more rarely, swallowing difficulties. The severity of such symptomatology depends on leukemic infiltration in CNS and consequently in its entity or increasing presence in CNS areas [6,7,8]. In addition, ALL appears underestimated due to underdiagnoses since it is asymptomatic in most cases. Diagnosis of ALL is based on the evaluation of the absolute number of all the circulating cells and the subsequent histopathological analysis of the marrow tissue. This last assessment is imperative and confirms the diagnosis thanks to the presence of 25% or more of the lymphoblasts in the bone marrow after the detailed examination of the composition and structure of the tissue sample and the use of appropriate techniques, i.e., cytogenetic methodology, and fluorescence in situ hybridization (FISH), for detecting typical chromosomal defects associated with different disease outcomes [9]. Anemia, leukopenia, and a low number of platelets explain the typical symptoms of ALL, including fatigue, weakness, shortness of breath during normal physical activities, light-headedness, dizziness or faintness, headaches, and a pale complexion, as well as recurrent fevers, frequent infections, easy bruising, red spots on the skin called petechiae, frequent or severe nosebleeds, bleeding gums, and, in women, more frequent menstrual periods. Additional symptoms include weight loss or loss of appetite, swollen glands, bone and joint pain, difficulty breathing, and an enlarged spleen or liver [10,11,12].

The ALL pathogenesis is evocated by both inherited and modulable factors, including genetic/chromosomic alterations and environmental factors. The latter category comprises alcohol, cigarettes, radiation exposure, chemical compounds, infections, and drugs. Modulable factors during pregnancy can affect the fetus by causing genetic mutations [13]. To date, however, the deep relationship between these factors and ALL onset, as well as the exact mechanisms and pathways involved in such complex ALL pathophysiology, are still not clear [14]. However, in the last few years, research has been focused on microbiomes since, in adult ALL cases, the microbiome has been demonstrated to be significantly associated with both onset and progression as well as modulation of the response and side effects of chemotherapy drugs, infection during treatment, and therapeutic efficacy [15,16,17]. Such particular attention is increasing given the growing evidence on the capacity of parents’ microbiomes and particularly of the gut microbiome (GM) to impact the formation, growth, and functions of a newborn’s hematopoietic system (HS) through fetal developmental programming as well as the newborn’s susceptibility to developing onco-hematological neoplasms [13]. Here, we describe and discuss the literature evidence on the GM role in pediatric ALL, as well as suggest some therapeutic strategies aimed at recovering microbiomes. 

## 2. Microbiome and ALL

In the last decade, recent evidence has demonstrated that another crucial determinant (trigger) of the development of several diseases is microbial dysfunction or, more accurately, dysbiosis. Dysbiosis has been shown to impact the development and progression of numerous human diseases, ranging from immune diseases, cardiovascular diseases, neurodegenerative diseases, and cancer [18,19,20,21,22]. The very relevant role of microbiota and microbiome (its genome) has led to their being considered as the second system of control of the functions of all the organs and tissues of the human body after the brain. Accordingly, the microbiome appears to be the second body of the brain, regulating both human health and disease. Among human diseases, microbial dysbiosis significantly contributes to cancer onset and progression [23,24], as evidenced for the first time in 2012 [25]. Research explorations on the association of GM and adult ALL started two years later [26], and investigations on childhood ALL were described only after 2016 [27]. It is likely that diverse issues have limited such studies. For instance, contamination of the tissue samples during the processing phase or during the entire assessment due to the use of contaminated buffers or other causes and inadequate processing techniques linked to high costs constitute the most common limitations reported. However, recent progress in omics technologies and the discounts in costs in such investigations, as well as the use of the appropriate animal models, such as the Pax5/- and Sca1-ETV6-Runx1 mice model, have facilitated the growing increase in the number of research studies on microbiomes in both adult and children leukemia [28,29]. Accordingly, the studies on Pax5/- and Sca1-ETV6-Runx1 mice have permitted the demonstration, for the first time, of the key role of genetic factors in contributing to affecting GM and how such variations can impact HS and leukemia development. This evidence suggests the consideration of GM analysis as a diagnostic leukemia tool in the future. In addition, interesting results from epidemiological studies have led to the supposition that a remarkable trigger of leukemia is infections, impacting the most common ALL subtypes. Also noteworthy are the results of a study conducted in 2018 on 42 pediatric patients with ALL at different times of therapy, revealing the presence of microbiomes, which vary depending on the type of cancer. Such findings, confirmed by another study conducted in 2020, have suggested that the human microbiome could be a diagnostic tool for specific types of cancer. Below, several concepts are clarified to better understand the close relationship between the microbiome and ALL in children [30,31,32].

## 3. Microbiota, Microbiomes, and Their Modulating Effects on Hematopoiesis in Health and Disease

Now, research interest in exploring microbiota is rising exponentially because of its key role in altering numerous mechanisms and processes related to human health and disease, such as cancer (e.g., ALL). Microbiota represents an important component of our body, constituted by many microorganisms (i.e., bacteria, viruses, and fungi). Precisely, microbial cells might be about 3.8 × 10^13^, with 3000 species. The microbiota, organized in niches, is localized in many anatomical structures of our body: oral, vagina, skin, and gut. However, the prevalent region of microbiota is the gut; consequently, here, it is defined as the gut microbiota (GM; as mentioned above), with 500 species of bacteria, as well as yeasts, parasites, and viruses. Regarding the bacterial species, the phyla *Firmicutes*, *Bacteroidetes*, and *Actinobacteria* are the most prevalent in adults [33], with *Firmicutes* including *Clostridium*, *Ruminococcus*, and *Eubacterium*. The bacterial species *Bacteroidetes* and *Firmicutes* show a crucial relationship both in health and disease [34]. 

**Figure 1:**
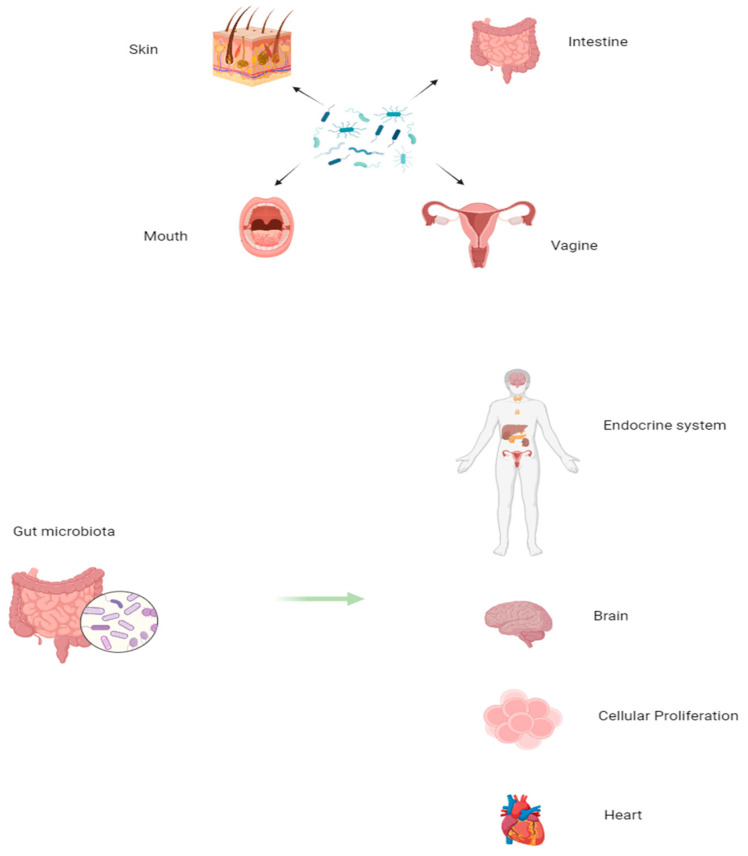
Presence of microbiota in the anatomical structures of our body and influence on different districts of our body.

In newborns, GM takes origin after birth, and the results are composed initially of native flora and later of transient flora derived by food intake [35]. Consequently, everybody has a specific and unique GM composition, which, however, changes during the duration of life, precisely from childhood to adulthood [36]. This results in a typical GM dynamism and plasticity, modulating the GM functions, as well as those of other tissues, organs, and systems, such as HS [37]. Therefore, GM influences, through multiple actions and effects, the functions of other tissues, all of which have a protective nature, contributing to the homeostasis of the organism. Precisely, GM contributes to protecting, through the body’s immune responses, the host from pathogens, and it has metabolic activities, contributes to the synthesis of amino acids, vitamins, and enzymes, and indirectly controls the proliferation, structure, development, and composition of cells, the immune system, and the intestine through the development of intestinal villi and intestinal epithelial cells, contributing to the formation of the epithelial barrier (see Figure 1).

In addition to these functions, GM also modulates the functions of the central nervous system, in which the metabolites released by the microbiota influence mood, stress regulation, and instinctive behaviors on the cardiovascular and endocrine–metabolic systems; the cardiovascular system is also modulated by GM through disease risk factors that cause hyperlipidemia, atherosclerosis, and type 2 diabetes and the digestive system through the action of microbiota products absorbed from the intestine and transferred to the liver (see Figure 1) [38]. Thus, GM also impacts the individual’s physical and mental health. However, even if generally considered beneficial, some GM microbes can cause a potential danger to the human organism [37]. When the homeostasis of the microbiota is disturbed, most of it becomes pathogenic, in which remodeling occurs that alters the sophisticated balance of the microecosystem in the gastrointestinal tract, contributing to a variety of pathological conditions and negatively affecting the physiological processes of the host [38]. This imbalance, called “microbial dysbiosis”, shows a reduction in microbes favorable to the health of the host and an excessive presence of pathogenic microbes with deleterious functions [39]. For example, *Bacteroides fragilis* and *Fusobacterium nucleatum* are known to induce a proinflammatory state in the colon, which could potentially increase oncogenic proliferation in the mucosal microenvironment [40,41].

Many risk factors can act on our microbiota; the first is immutable and is genetically inherited; it cannot be modified; however, other factors can instead be modified, such as the environment and the way we are born and nourished during the first 1000 days of life, as well as the programming of the development of all organs, such as HS, during fetal life, depending on the parents, mother, and father [42]. Malnutrition or overnutrition in pregnancy is involved in influencing fetal programming, just as other maternal and paternal factors modulate fetal programming. The components of the intestinal flora are strongly dependent on the influence of the environment in which we live, such as rural, urban, industrialized, and non-industrialized areas [43]. Dysbiosis can inhibit the colonization of beneficial probiotic bacteria, promote the proliferation of harmful enteropathogens, and alter innate receptors and cytokine signaling, thereby affecting the immune system [44]. Dysbiosis can contribute to and be associated with a myriad of pathological conditions, such as inflammatory bowel diseases, immune-mediated diseases, and neoplastic conditions, including hematological malignancies [39].

### 3.1. The GM’s Close Relationship with Hematopoiesis

Recent evidence reports that GM has a close relationship with hematopoiesis. Accordingly, the Balmer group first reported in 2014 that the diverse composition of the microbiota significantly influences both the configuration and quantity of myeloid cell lineages [45]. This could explain why the microbiota with fewer types of microorganisms constitutes an unfavorable factor for the success of an allogeneic stem cell transplant, as highlighted by Taur and collaborators. It has, in fact, been shown that this unfavorable condition is significantly correlated with an increase in mortality and consequent lower survival rates of transplanted patients [46]. Similar results were found in mice treated with antibiotic therapy [47]. Furthermore, it has been reported in mice that some foods are able to impact the microbiota and can indirectly modify the function of HS. For example, foods rich in fiber specifically modify the function of some bacteria in the microbiota, capable of transforming these foods into short-chain fatty acids (SCFA). Therefore, mice fed a diet rich in fiber-containing foods were found to have higher levels of myeloid precursors in the bone marrow [48]. Similarly, high systemic levels of lipopolysaccharides related to GM dysbiosis have been shown to induce myelosuppressive effects through the chronic activation of the innate immune Toll-like receptor expressed in hematopoietic stem cells (HSCs) [49]. These observations suggest that the relationship between alterations in the microbiota and the onset of numerous immune disorders is linked to the development of age-related diseases.

As reported above, malnutrition or overnutrition in pregnancy influences the programming of fetal development. Furthermore, growing evidence shows the role of environmental experiences in influencing the gut microbiota early in life, underlining a “microbial programming phenomenon” (widely cited in [50]). In this context, obesity has emerged as a significant health challenge for both mothers and children. In children, obesity affects the development of both the microbiota and immune responses. Despite its importance, it is unclear whether weight during pregnancy, as well as the composition and functional quality of the maternal microbiota, can mediate adverse effects on offspring. However, Kozyrskyj and colleagues reviewed literature from human studies and determined that maternal obesity can modulate both the composition and function of the gut microbiota in the newborn [51]. The vertical transport of the microbiota and the release or inhibition of the release of their metabolic products have been hypothesized as possible mechanisms [52].

In the next paragraphs, the GM’s effects on hematopoiesis are better described, as well as the role of developmental programming of HS. 

### 3.2. Experimental Evidence for the Effects of Microbiota on Hematopoiesis

Research on the topic of GM is still in the primary development phase; however, there are some encouraging results regarding the relationship between GM and hematopoiesis. In this context, it has been shown that different GM products affect hematopoiesis, and the composition of the bone marrow cell pool is significantly correlated with the structure and heterogeneity of GM [53]. Studies on mice are of substantial help because they contribute significantly to the identification of the effects of GMOs on HS. Studies on germ-free mice have highlighted reduced immunological functions. This altered phenotype has been shown to be associated with increased susceptibility to intestinal and systemic infections [54]. These results highlight the value of GM functions. As reported above, GMs can carry out numerous and specific biochemical and molecular reactions and consequently synthesize a broad spectrum of molecules and metabolites that human cells are unable to produce. Some GM compounds exert an effect on hematopoietic functions. For example, SCFAs interact with the immune system through G protein-coupled receptors (GPCRs) expressed in many immunological cells. In many cases, SCFAs mediate an anti-inflammatory effect through the inactivation of the NF-kB pathway. From a hematopoietic point of view, SCFAs promote the generation of specific subsets of T cells [55,56]. In another study on mice, the impact of the use of fermentable fibers as a dietary supplement on the immune system was analyzed. The results reported that increasing fiber bioavailability leads to the expansion of members of the Bacteroidetes phylum. In turn, this results in a significant local (intestinal) and systemic increase in SCFAs. Enhanced SCFA concentration enhances the proliferation of dendritic cell precursors in the bone marrow through the activation of GPR41. An interesting fact is that GM variations are accompanied by alterations in the lung microbiota, even if they do not cause an increase in SCFA levels, probably due to the lack of suitable substrates [57]. As mentioned above, antibiotic treatments often cause severe hematopoietic damage through the suppression of its activities. This condition is not caused by the direct effect of antibiotic drugs. Accordingly, significant data were obtained by Josefsdottir and collaborators [58]. They explored the harmful effects of antibiotics by depleting GM mice through antibiotic treatment. GM depletion in mice results in the substantial impairment of hematopoietic function, including a reduction in the number of cells in the blood related to decreased cellularity in the bone marrow. In the study, they determined that GMO depletion, and not antibiotic treatment, caused this acquired hematopoietic phenotype. GMO transplantation has indeed been shown to restore physiological hematopoiesis. They observed that immunological deterioration was linked to antibiotic-induced GM alterations, causing an immune phenotype identical to that of Stat-1-deficient mice. This has led to the assumption that the suppression of Stat-1 signaling induced by GM suppression is systemic and not limited to the intestinal area [59]. The global findings mentioned above are far-reaching when considering the plasticity and variability of GM. The GM is extremely vulnerable to environmental and nutritional factors, as demonstrated in twin studies [60,61]. Furthermore, GM alterations are correlated with age, although some specific GM characteristics of centenarians have been related to the longevity phenotype [61,62,63,64,65]. Taken together, the great connection between microbiota and hematopoiesis constitutes a significant new factor that is capable of modulating the fate of individual immuno-biographical traits [62] in health and disease [65].

### 3.3. Developmental Programming of HS and the Susceptibility of Leukemia

Well-established evidence has recognized that human body systems, such as the nervous, endocrine, immune, and cardiovascular systems, have been programmed during fetal development [66,67,68,69,70,71,72,73,74,75]. The fetal period (and especially the embryonic stages; “critical windows”) is highly susceptible to many environmental stressors, which can impact life after birth, the adult period, and its health status [76]. Therefore, it characterizes an essential period of development, characterized by a high rate of cell proliferation and plasticity in developing systems. These observations are significantly associated with new concepts on the high vulnerability of our systems to maternal, environmental, and intrauterine stressors during their embryonic development [50,66,67,68,69,70,71,72,73,74,75]. Harmful developmental conditions can influence the (epi)genetic and physiological processes of fetal development, thus permanently modifying the construction and functionality of the hypothalamic–pituitary–adrenal (HPA) axis and offspring systems, predisposing them to the development of disease during life after birth [50,66,67,68,69,70,71,72,73,74,75]. All concepts illustrated imply that the function, fate, and disease of the HS in postnatal life clearly depend on developmental programming processes. Endogenous and gestational risk factors have been shown to influence this process, its mechanisms, and induced effects on the HPA axis, immune, endocrine, and metabolic systems, inducing developmental programming of HS and outcomes in life after birth [13]. Overall, these concepts have led to interesting evidence on the critical relevance of the close relationship between the maternal and fetal microenvironment and its significant contribution to programming on the development of HS and susceptibility to diseases, such as leukemia [66,67,68,69,70,71,72,73,74,75,76,77]. Consequently, some studies have reported that prematurity at birth and intrauterine growth restriction (IUGR) may be major risk factors for HS alterations, contributing to the increased HS susceptibility to HS diseases, such as leukemia [66,67,68,69,70,71,72,73,74,75,76,77].

## 4. Close Relationship between ALL and Microbiota

**Figure 2:**
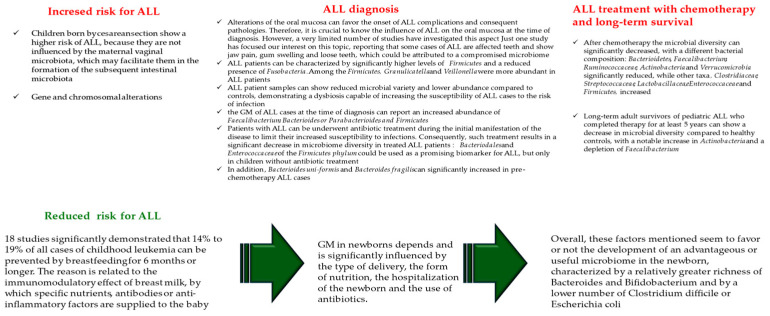
Alterations of microbiome and its relationship in all the phase of ALL and protective factors for a microbiome in health with reduced risk for ALL.

Recent evidence reports microbiome alterations in children with ALL. They have been observed in all the steps of ALL management: at both the time of diagnosis, during treatment, and in contributing to the onset of complications and differences observed after the completion of therapy (see Figure 2). At the onset of disease, a small variation in the oral and gut microbiota/microbiome of ALL children patients is already detected [78]. During the ALL treatment, the administration of chemotherapeutics and antibiotics causes ulterior alterations in the microbiome by determining an additional reduction in the diversity of composition and types of the microbiota’s microorganisms. Such results in the dominance of *Enteroccocaceae* are predictive of infections. Furthermore, microbiota deterioration, including *Faecalibacterium* depletion, may extend for several years after completion of the ALL treatment, inducing potential long-term health effects. Nevertheless, it is not clear whether the observed alterations represent a fundamental trigger for the development of ALL or are caused by immunological variations that precede the onset of ALL. Monitoring large pediatric cohorts could be useful to provide direct evidence on whether genotypes already determine microbial composition, even without disease onset. Considering all this together, there is always a clear need for the precise characterization and modulation of patients’ microbiomes during therapy to better understand the microbial influence on leukemogenesis, minimize side effects, and improve treatment efficacy [78].

In the next paragraphs, we stress these concepts to better understand the messages of such a review. 

### 4.1. Gut Microbiota and Child Susceptibility for ALL Onset

The role of GM is evident from the birth of newborns, and its composition and passage from mother to child can significantly influence the onset and type of cancer. For example, children born by a cesarean section show a higher risk of ALL because they are not influenced by the maternal vaginal microbiota, which may facilitate them in the formation of the subsequent intestinal microbiota [78]. However, two recent studies have reported a non-association between the type of delivery and the occurrence of brain cancer or lymphoma [1,79]. Another study, namely a meta-analysis conducted on 25 studies, 18 of which met all the inclusion criteria, has evaluated the association between breastfeeding and the onset of ALL. All subgroup meta-analyses of the 18 studies significantly demonstrated that 14% to 19% of all cases of childhood leukemia can be prevented by breastfeeding for 6 months or longer. The reason is related to the immunomodulatory effect of breast milk, by which specific nutrients, antibodies, or anti-inflammatory factors are supplied to the baby [80]. Based on such interesting findings, it is suggested that early GM in newborns significantly depends on and is influenced by the type of delivery, form of feeding, hospitalization of the newborn, and the use of antibiotics. Overall, these factors mentioned seem to favor or not the development of an advantageous or useful microbiome in the newborn, characterized by a relatively greater richness of Bacteroides and Bifidobacterium and by a lower number of Clostridium difficile or Escherichia coli [81] (see Figure 2).

### 4.2. Alterations in Microbiome at the Children ALL Diagnosis

As reported above, microbiome alterations can affect both the oral and gut microbiomes. The oral mucosa constitutes the first natural protective barrier, and its alterations can favor the onset of ALL complications and consequent pathologies. Therefore, it is imperative to know the influence of ALL on the oral mucosa at the time of diagnosis and during chemotherapy. However, a very limited number of studies have investigated this aspect. Precisely, one study focused our interest on this issue, reporting that some cases of ALL affect the teeth and cause jaw pain, gingival swelling, and loose teeth, which could be attributed to a compromised microbiome [30,82]. Analysis of oral samples from newly diagnosed ALL patients, compared with healthy control children, demonstrated that *Firmicutes* and *Fusobacteria* were significantly different. Precisely, ALL patients were characterized by significantly higher levels of *Firmicutes* and a reduced presence of *Fusobacteria.* Among the *Firmicutes*, *Granulicatella* and *Veillonella* were more abundant in ALL patients [82]. Taken together, ALL patient samples showed reduced microbial variety and lower abundance compared to controls, demonstrating a dysbiosis capable of increasing the susceptibility of ALL cases to the risk of infection. Certainly, further studies will have to confirm this aspect. Investigations of the GM of ALL cases at the time of diagnosis reported an increased abundance of *Faecalibacterium*, *Bacterioides* or *Parabacterioides*, and *Firmicutes* [83,84]. However, diarrhea-causing *Clostridium* has been observed to be less abundant in ALL children, whereas *Lachnospiraceae*, including *Roseburia* and *Blautia*, produce SCFAs with an anti-inflammatory effect [85]. Despite this, some patients with ALL underwent antibiotic treatment during the initial manifestation of the disease to limit their increased susceptibility to infections. Consequently, such treatment results in a significant decrease in microbiome diversity in treated ALL patients [84]. Based on these findings, Bai and colleagues suggested that *Bacteriodales* and *Enterococcaceae* of the *Firmicutes phylum* could be used as a promising biomarker for ALL but only in children without antibiotic treatment [84]. In 2020, Liu and colleagues, on the largest cohort of cases examined so far, namely 70 patients with newly diagnosed ALL enrolled at the time of diagnosis [83], did not observe any difference in the alpha diversity of the microbiome and confirmed an increase in the *Bacteroides* species; therefore, in beta diversity. Specifically, *Bacterioides uni-formis* and *Bacteroides fragilis* significantly increased in pre-chemotherapy ALL cases (see Figure 2).

### 4.3. Alterations in Microbiome during Children ALL Treatment with Chemotherapy

Treatment of ALL with chemotherapy begins immediately after diagnosis and is characterized by three phases over 2–3 years. Such treatment is known to change bacterial composition and cause a shift in the reduction in white blood cell counts. This leads to the development of symptoms typical of childhood ALL, including fatigue resulting from anemia, fever, infections, and even easy bleeding. Therapy begins immediately after diagnosis, with chemotherapy administered in three phases over 2–3 years. Furthermore, infections are characteristic. Consequently, a study conducted on 409 patients with newly diagnosed ALL demonstrated the presence of 1313 infections of microbiological origin during therapy [86]. This has led to the hypothesis of the role of the altered gut microbiome on the significant increase in susceptibility to documented infections. On the other hand, one study showed that the composition of the microbiome changes when compared before and after treatment, with a specific reduction in *Lachnospiraceae* and *Roseburia* in patients [87,88]. A larger study by Hakim and colleagues of 199 ALL patients conducted during three different chemotherapy phases showed no significant differences in the average variety of the microbiome, which showed baseline characteristics; however, after chemotherapy, the microbial diversity significantly decreased, with a different bacterial composition: *Bacterioidetes*, *Faecalibacterium*, *Ruminococcaceae*, *Actinobacteria*, and *Verrucomicrobia* significantly reduced, while other taxa, *Clostridiaceae*, *Streptococcaceae*, *Lactobacillaceae*, *Enterococcaceae*, and *Firmicutes*, increased [87].

However, a longitudinal observational study conducted by Chua and colleagues analyzed temporal differences in the GM of seven ALL patients before, during, and after chemotherapy and compared them to controls [88]. They observed that antibiotic treatment before chemotherapy was not the cause of the observed differences in the large interindividual variability of ALL patients compared to healthy children in microbiome composition. However, *Bacteroidetes* were found to be significantly enhanced before chemotherapy, although their large quantity decreased after therapy. In contrast, *Firmicutes* and *Actinobacteria* increased after chemotherapy to a similar level compared to healthy controls. Five genera precisely were found to have a lower abundance after therapy: *Bacteroides* and *Prevotella*, belonging to the phylum *Bacteroidetes*; *Fusobacterium*; and *Atopobium* and Corynebacterium from *Fusobacteria* and *Actinobacteria*, respectively. Furthermore, only *Bifidobacterium (Actinobacteria)* was significantly higher in post-chemotherapy samples. This commensal occurs immediately after birth and can utilize human milk oligosaccharides as well as other carbon sources. Furthermore, it has a protective role in preventing intestinal inflammation in newborns as it prevents an increase in *Proteobacteria* associated with dysbiosis and negative health outcomes [86,89]. 

Finally, long-term adult survivors of pediatric ALL who completed therapy for at least 5 years showed a decrease in microbial diversity compared to healthy controls, with a notable increase in *Actinobacteria* and a depletion in *Faecalibacterium* [88]. Furthermore, researchers detected increased T-cell activation and chronic inflammation in these individuals, suggesting a correlation between dysregulated microbial taxa and immune dysregulation [88]. The increased risk of infection in survivors, a high prevalence of chronic health conditions, and a high risk of mortality and morbidity have also been studied [90]. Therefore, microbial dysregulation brought about by the effects of chemotherapy and antibiotics during the treatment of ALL may have long-term effects on the development of other diseases, such as obesity or diabetes, in adult survivors of pediatric ALL [91,92] (see Figure 2).

## 5. Diverse Levels of Strategies to Recovery Microbiome and Prevent ALL Onset

### 5.1. The First Level of Health-Promoting Strategies

**Figure 3:**
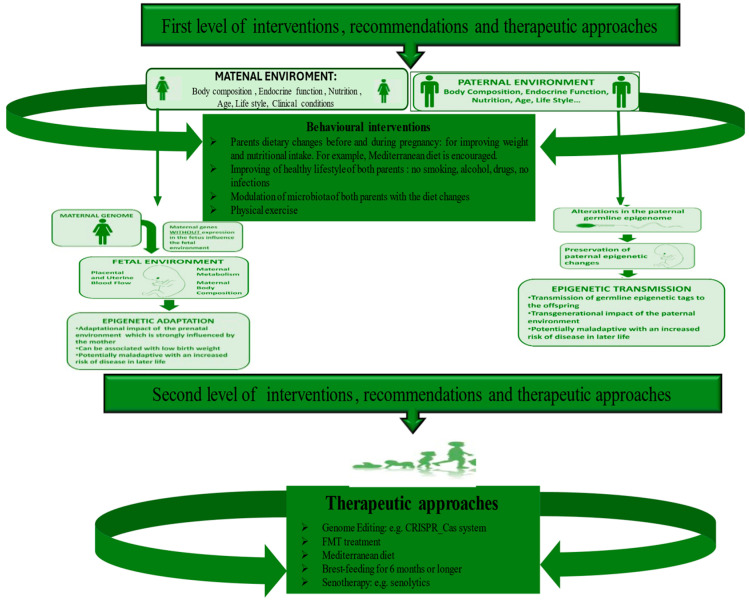
Diverse levels of strategies to recovery microbiome and prevent all onset.

The microbiome is crucial to an individual’s life and, compared to other systems, shows interindividual variation, particularly in ALL cases. Today, growing evidence suggests that these differences do not originate in the later stages of an individual’s life cycle but rather in the early periods. The latter constitute critical windows of rapid growth under strong epigenetic remodeling and, therefore, show high dynamism and phenotypic plasticity, during which an organism is predominantly susceptible to environmental conditions, harmfully influencing the development of tissues, organs, and systems and the tendency to illnesses later in life or a few years after birth. A clear example is the ALL cases (widely cited in [50,93]). We have reported here how the HS is evolutionarily programmed, like other systems, and, therefore, in adulthood or a few years after birth, it is the representation of evolutionary programming and, in particular, the consequence of numerous evolutionary programming events. Among these, the (re)programming of the endothelium is included first, which is the core of the origin, homeostasis, and function of the HS, as well as other human systems [94,95,96,97]. In turn, the (re)programming of the endothelium influences HS and immune programming with the cooperation of hormonal and metabolic alterations (altered HPA axis and increased release of cortisol, but also other crucial hormones). Subsequently, they induce epigenetic and microbial programming in the offspring. The combination of all these programming conditions makes the HS of the offspring susceptible to assuming long-term structural and functional alterations, which permanently modulate its functions and increase the risk of diseases and the speed of aging. However, their prevention, as well as the prevention of consequent pathologies with long or short onset (i.e., ALL), may be feasible and consequently represent a certainty and not an illusion. Accordingly, evidence supports the positive effects of a healthy diet (e.g., Mediterranean), physical activity, low stress, non-smoking, alcohol, and drug-free use during pregnancy [98,99,100,101,102]. Here, we first suggest recommending such strategies to both parents who wish to have children before and during pregnancy. McGowan and Matthews, in 2018, supported such strategies, emphasizing the role of both parents and their lifestyles in developmental planning. Precisely, they state that parental danger (not only maternal but also paternal) is linked to stress, and/or their changed clinical status is linked to being affected by pathologies (e.g., hypertension and type 2 diabetes) or having an unhealthy lifestyle. The lifestyle (linked to alcohol consumption, the use of drugs, diet, or being a smoker and sedentary) causes profound biological effects both on fetal development and on the subsequent functionality of the HPA axis and specific systems. Furthermore, these effects appear to be species-, genus-, and age-specific and vary depending on the timing and duration of exposure, as highlighted by McGowan and Matthews (2018) [74] (see Figure 3).

### 5.2. Some Therapeutic Approaches of Second Level

In addition to first-level health promotion strategies, some second-level therapeutic approaches should be applied during the prenatal and neonatal period or in adult life (see Figure 3). Among these, importance was given to the pharmacological objectives/targeting of the pathways involved in endothelium-HS crosstalk, cellular/tissue reprogramming, the use of miRNAs, the modulation of the microbiota of both parents and newborns through the innovative method of fecal examinations, and microbiota transplantations (FMTs) [103]. Precisely, FMT is a promising treatment for diseases related to intestinal dysbiosis since it can help rebalance the composition and function of the intestinal microbiota by transferring fecal preparations from healthy donors [104]. The effectiveness of the treatment can be explained by considering, for example, the metabolites derived from the Firmicutes phylum, in particular SCFAs and secondary bile acids, which, with their beneficial roles such as fortifying the intestinal barrier and alleviating inflammation, promote host homeostasis [105]. FMT can directly modify the recipient’s GM to normalize composition and provide therapeutic benefits. FMT was initially applied for the treatment of recurrent and refractory Clostridium difficile infections thanks to the decisive consensus of the US Food and Drug Administration in 2013. Today, its application is not only limited to gastrointestinal disorders but to other diseases [103]. Accordingly, a recent systematic review highlighted that FMT can be adopted for the treatment of 85 specific diseases in clinical settings globally from 2011 to 2021 [106]. Furthermore, a study conducted at the University of Minnesota, enrolling patients suffering from acute myeloid leukemia and patients undergoing hematopoietic cell transplantation, experimented with microbiota transplantation. The enrolled subjects were fragile and could contract a high number of infections, which is also linked to alterations in the microbiota (dysbiosis) following the treatment. Although microbiota transplantation does not have a significant effect against infections, it can still normalize the composition of the microbiota, obtaining therapeutic benefits by improving the diversity of intestinal microorganisms, increasing the levels of some anaerobic commensal bacteria, and reducing the concentration of other species that could be the cause of some disorders [107].

Although FMT appears to be a generally safe therapeutic method with few adverse effects, it is nevertheless necessary to monitor clinical efficacy and long-term adverse events [103]. Consequently, it is imperative to establish regular follow-ups to identify the periodicity and duration of the FMT treatment and monitor clinical efficacy and long-term adverse events. Furthermore, further studies are needed to develop personalized FMT treatments for each individual and their clinical conditions based on different characteristics of the host and the diseases/conditions to be treated, such as adverse programming of HS and leukemia.

Another strategy could be the use of serotherapy, which includes three therapeutic approaches: (i) treatment with molecules capable of selectively killing senescent cells (SC), i.e., sanolytics; (ii) the use of compounds that have the ability to reduce the proinflammatory program of SCs or which modify the senescent phenotype, i.e., mammals; and (iii) prevention of the accumulation of senescent cells [108,109,110,111]. All these measures could allow (a) a reduction in the effects of adversity on development, (b) favoring well-matched developmental programming of HS that acts mainly on the endothelium, and (c) the delay/delay onset of leukemia in children and in adulthood. Additionally, the development of other optional treatments is increasing. The latter has the aim of recovering the disturbed epigenetic profiles linked to altered programming. However, harmful epigenetic alterations are believed to be potentially reversible; therefore, they could be corrected by some lifestyle factors such as diet and physical activity as well as by pharmacological interventions specifically targeting the epigenome [112,113,114,115]. If these therapeutic strategies are established, then such an approach would provide a way to slow the epigenetic clock and modify epigenetic age dynamics throughout the life course and, therefore, slow and/or delay age-related changes and processes [93,116,117]. The lack of specific biomarkers to monitor developmental programming makes it difficult to test and verify the biological effects of possible interventions and treatments. Goswami’s group suggested telomere length as an optimal biomarker for developmental programming [118]. Epigenetic indices of age, such as DNA methylation-based biomarkers, are also now considered another promising option [119]. These obstacles, as well as the need to identify unknown long-term outcomes of the interventions and therapeutic approaches described, reflect several gaps and the need for further studies. Multidisciplinary investigations are particularly suggested, all being the result of the sophisticated interaction of environmental factors with its genome, transcriptome, proteome, metabolome, microbiome, epigenome, and exposome, as highlighted in the description and discussion on HS programming. Further studies are, therefore, essential for different types of patients with different conditions and diseases [103].

Diet plays a key role from the first days of life in human health in cellular metabolism, GM regulation, and immunological processes via epigenomic factors, as mentioned above [120]. Many studies have reported a correlation between habits such as smoking and drinking alcohol during pregnancy and an increased risk of leukemia, but other dietary factors also have an important influence [121,122]. Fruit and vegetable intake provides the folic acid necessary to avoid the risk of leukemia in children, and it has been reported that maternal fruit and vegetable consumption is inversely related to childhood ALL [123]. In California, according to these premises, a study was conducted to evaluate the link between the quality of the maternal diet before pregnancy, considering a diet quality index, and the risk of childhood ALL. On the other hand, maternal malnutrition and low levels of micronutrients could cause elevated maternal cortisol concentrations, affecting the development of the fetal immune system and interfering with normal immune cell proliferation and organogenesis [124]. Studies have reported a correlation between age and ALL risk. It has been noted by some researchers that a diet that includes fish, seafood, beans, and beef is related to a low risk of ALL [125]. Instead, the risk of ALL may increase when mothers consume various foods such as sugars or syrups [126]. Regarding the risk of ALL in children, there is a positive association between the risk of this disease and the consumption of more coffee and/or caffeinated drinks [127,128].

Dietary habits can influence the diversity of GM, and food components can influence both the microbial population and its metabolic activity. The Mediterranean diet has been proposed, which includes foods such as vegetables, fiber, omega-3 fatty acids, animal proteins, and saturated fats but in smaller quantities. Adherence to the Mediterranean diet leads to an increase in some bacteria such as *Bifidobacteria*, *Lactobacilli*, *Prevotella*, *Eubacteria*, and *Bacteroides*; on the contrary, a diet rich in fat leads to an increase in Bacteroides and Enterobacteria and a decrease in *Bifidobacteria*, *Lactobacilli*, *Prevotella,* and *Eubacteria*. Several studies affirm this diet-dependent change in the microbiota [129]. In a study by De Filippis and colleagues, in 153 individuals who habitually followed omnivorous, vegetarian, or vegan diets, it was observed that adherence to this diet was associated with an increase in the levels of SCFA (short-chain fatty acids) degrading fibers, *Prevotella* and *Firmicutes* [130]. In subjects following the Mediterranean diet, the *Prevotella–Bacteroides* ratio was higher, indicating that a diet rich in natural fiber and resistant starch has a positive effect on the bacterial composition of human subjects [131]. A study conducted by Garcia’s group focused on the eating habits of healthy subjects to test the variability of the microbiota adhering to the Mediterranean diet. What is observed following a questionnaire to which volunteer subjects are subjected is that adhering to the Mediterranean diet allows a decrease in the *Firmicutes–Bacteroidetes* ratio and a greater presence of *Bacteroidetes* was associated with a lower intake of animal proteins. The high consumption of animal proteins, saturated fats, and sugars influenced the diversity of the intestinal microbiota [132]. To confirm the observations of the above studies, a preclinical study conducted on mice by Nagpal et al. analyzed the gut microbiome after adhering to the typical Western diet or the Mediterranean diet [133]. They found that the microbiome of study participants consuming the typical Western diet was significantly more diverse than the microbiome of participants consuming the typical Mediterranean diet. They found that the microbiota of study participants consuming the Mediterranean diet was significantly more diverse than the microbiota of participants consuming the Western diet, characterized by lard, beef tallow, butter, eggs, cholesterol, casein, lactalbumin, dextrin, corn, high fructose syrup, and sucrose. They also had a higher abundance of *Lactobacillus*, *Clostridium*, *Faecalibacterium,* and *Oscillospira* and a lower abundance of *Ruminococcus* and *Coprococcus* [133]. Another study states that diet can alter the composition of the microbiota very quickly, in less than a week, as demonstrated by 31 subjects in his study, which states that the consumption of certain types of food produces predictable changes in existing bacterial host genera. This influences host immune and metabolic parameters, with broad implications for human health [129]. Microbes in the distal intestine, where they are abundant, contribute to host health through the biosynthesis of essential vitamins and amino acids and the generation of important metabolic byproducts from food components undigested by the small intestine [134].

Numerous studies have been able to comprehensively investigate the impact of the food component on the intestinal microbial composition. The effects of dietary protein on the gut microbiota were first described in 1977. Protein consumption is positively correlated with overall microbial diversity [135]. Consumption of whey and pea protein extract leads to an increase in intestinal *Bifdobacterium* and *Lactobacillus*, while whey also reduces the pathogens *Bacteroides fragilis* and *Clostridium perfrigens* [136]. Pea protein has also been associated with increased levels of intestinal SCFAs, which are considered anti-inflammatory and important for maintaining the mucosal barrier [137]. In contrast, consumption of animal proteins causes an increase in the number of bile-tolerant anaerobes, such as *Bacteroides*, *Alistipes*, and *Bilophila* [135]. One study found that subjects following a high-protein, low-carbohydrate diet had a reduced presence of *Roseburia* and *Eubacterium rectale* in the gut microbiota and a low level of butyrate in the stool [138]. In their study, De Filippo et al. observed lower fecal SCFAs in Italian subjects consuming a high-protein diet [139]. It has also been shown that adhering to the Mediterranean diet has positive effects on health, with a reduction in inflammatory molecules and, therefore, a protective role against oncological diseases.

Regarding the panel of markers linked to inflammation, a study was conducted examining the effects of the Mediterranean diet on the inflammatory profile. A total of 612 non-frail or pre-frail subjects in five European countries (UK, France, Netherlands, Italy, and Poland) were analyzed before and after a 12-month Mediterranean diet intervention. After adherence to the diet, a negative correlation was observed between the inflammatory markers CRP, IL-17, and IL-2, with positive levels of the anti-inflammatory cytokine IL-10. This confirms how diet and, in particular, adherence to the Mediterranean diet can positively influence health by reducing the risk of chronic inflammatory diseases [140]. In addition to their effects on the composition of the microbiota, prebiotics also produce significant changes in metabolic and immune markers. Several studies have observed reductions in the proinflammatory cytokine IL-6, which is associated with the intake of non-digestible carbohydrates present in whole grains [141]. West et al. noted increased plasma levels of the anti-inflammatory cytokine IL-10 with the consumption of high-amylose cornstarch butyrate [142]. Therefore, prebiotics are thought to have a beneficial effect on the immune and metabolic function of the gut, and this is believed to be due to increased SCFA production and the strengthening of gastrointestinal tract-associated lymphoid tissue (GALT) resulting from the fermentation of the fibers [143]. New evidence has reported that specific nutrients exert different actions on metabolic outcomes, depending on individual microbial patterns subject to specific individuals or conditions, suggesting the important role of personalized human nutritional treatment [144]. Another recent therapy is based on the use of butyrate-producing bacteria, which, as previously mentioned, has a protective role against ALL diseases. These strains, such as those of *Clostridium butyricum* and *Butyricicoccus pullicaecorum*, are believed to be specific next-generation niche probiotics and have good bile tolerance, viability, and metabolism and can be genetically manipulated to increase their ability to produce butyrate [145,146]. For example, heterologous genes required for butyrate production from acetyl-CoA butyrate can be introduced by inactivating the gene encoding the conversion of acetyl-CoA to acetate and the gene encoding the aldehyde/alcohol dehydrogenase for ethanol production or simply by disrupting a CoA transferase gene, which could be an alternative route for acetate production. Furthermore, to obtain higher levels of butyrate and, therefore, a greater abundance of butyrate producers in the intestine, it is possible to carry out a co-culture to obtain an interactive microbial population composed of more than just microbes.

## 6. Conclusions

**Figure 4:**
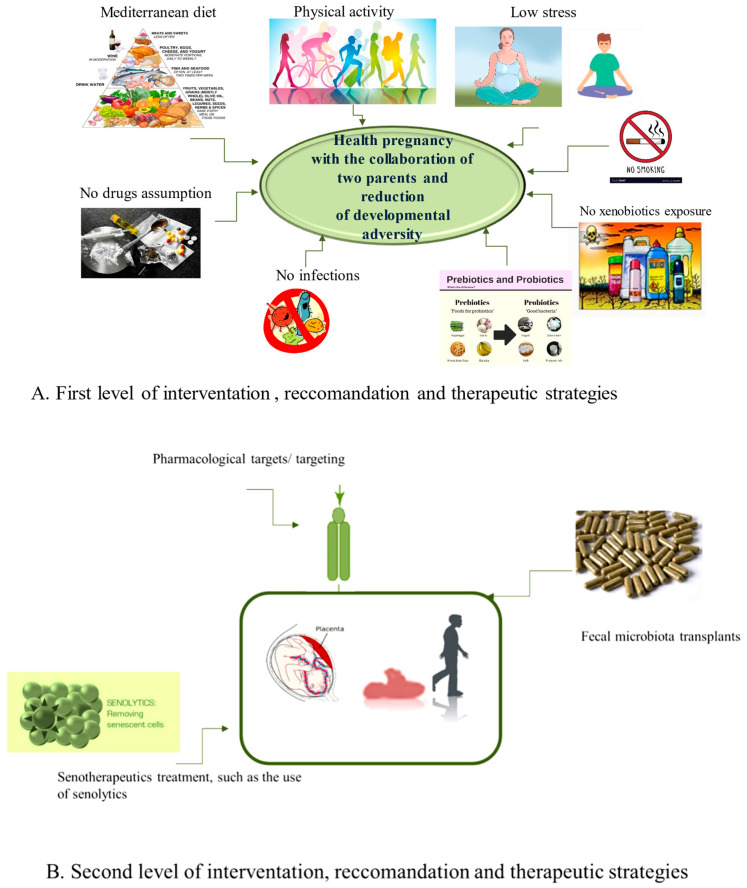
Two levels of interventions, recommendations, and therapeutical strategies in parents (**A**) and in children after the birth (**B**).

Although studies on GM are growing in number, the correlation between pediatric cancers, such as ALL and GM, is not yet well recognized. It is complicated to study due to the relatively small number of patients (cohorts) and difficulties in sample collection. Additionally, children with ALL receive chemotherapy and radiation therapy as well as intensive antibiotic prophylaxis to inhibit potential infections. These treatments have a great effect on the entire body, including the bone marrow, liver, and gastrointestinal tract, and directly and indirectly affect the GM. Furthermore, no prospective study has been designed for ALL to date. The relevance of GM is confirmed by numerous studies regarding the development of the disease, the effectiveness of therapy, the staging, and the manifestations of side effects. It requires further investigation, but in the future, it may be possible to identify an individual’s microbiome profile before starting cancer therapy to predict its effectiveness or choose an appropriate and personalized therapy. GM could also be used as a biomarker. Whether dysbiosis is a consequence or a cause of neoplasms remains unanswered. Studies on the microbiome in the pediatric oncology population are limited, and associations are not yet clear. Further studies with larger cohorts are needed to help develop more personalized and successful therapy in pediatric oncology. Finally, further studies are essential to test the long-term effects of the therapeutic strategies that are suggested to be applied at two levels of intervention (see Figure 4A,B).
